# Case report: Umbilical vessel aneurysm thrombosis and factor V Leiden mutation leading to fetal demise

**DOI:** 10.3389/fmed.2022.1083806

**Published:** 2023-01-04

**Authors:** Camélia Oualiken, Olivia Martz, Nadia Idrissi, Fara Tanjona Harizay, Laurent Martin, Emmanuel De Maistre, Lou Ricaud, Georges Tarris

**Affiliations:** ^1^Department of Pathology, University Hospital of Dijon, Dijon, France; ^2^Forensics Institute, University Hospital of Dijon, Dijon, France; ^3^Department of Obstetrics and Gynecology, Prenatal Diagnostic Center, Gynecology Emergency Services, University Hospital of Dijon, Dijon, France; ^4^Private Practice Center, Dijon, France; ^5^Department of Hemostasis, University Hospital of Dijon, Dijon, France

**Keywords:** umbilical vessel aneurysm, thrombosis, thrombophilia, fetal demise, stillbirth, umbilical artery aneurysm

## Abstract

Complicated pregnancies are nowadays a major public health concern, with possible lethality or sequelae both for the mother and the fetus. Blood coagulation disorders (including antiphospholipid syndrome, factor V Leiden mutation and antithrombin deficiency) and hypertensive gestational disorders are very well-known contributors of complicated pregnancies with poor fetal outcome, such as intrauterine growth retardation (IUGR) and fetal demise. Less commonly, vascular malformations of the placenta can also potentially lead to serious complications such as IUGR and fetal death. These malformations include hypercoiled umbilical cord, umbilical cord knot, umbilical cord varix, umbilical cord arterial or venous aneurysm, and velamentous insertion of the umbilical cord potentially leading to Benckiser's hemorrhage. Here, we report the case of a 29-year-old Gravida 2 Para 0 mother with previous history of stillbirth and smoking, admitted to the obstetrics department for the absence of fetal movement at 38 weeks of amenorrhea (WA). First-trimester and second-trimester routine ultrasounds were otherwise normal. Ultrasound performed at 38 WA revealed a 83 × 66 × 54 mm cystic heterogenous mass at the umbilical cord insertion. After delivery, fetal and placental pathology as well as maternal blood testing were performed. Fetal pathology was otherwise normal, except for diffuse congestion and meconial overload suggesting acute fetal distress. Fetal karyotype was normal (46 XX). Placental pathology revealed an umbilical artery aneurysm (UAA) at the base of the insertion of the umbilical cord, lined with a CD34^+^ CD31^+^ endothelium. After dissection, the aneurysm was filled with hemorrhagic debris, indicating aneurysm thrombosis. Histopathology revealed associated maternal vascular malperfusion (MVM) and increased peri-villous fibrin (IPF). Maternal blood tests revealed heterozygous factor V Leiden mutation, without other associated auto-immune conditions (such as antiphospholipid syndrome). Umbilical artery aneurysms remain extremely rare findings in the placenta, with <20 reported cases. Umbilical artery aneurysms have tendency to be located at the base of the insertion of the placenta, and lead to fetal demise in more than 60% of cases, mainly due to aneurysmal thrombosis, hematoma, possible vascular compression and/or rupture. Umbilical vessel aneurysms can be associated with trisomy 18 or 13. In our case, the association of factor V Leiden mutation, a hypercoagulable state, with UAA could explain massive thrombosis of the aneurysmal lumen and sudden fetal demise. Further consideration of current guidelines for surveillance and management of UAA would allow appropriate planned delivery in maternal care settings.

## 1. Introduction

Fetal demise remains a major concern in the course of a pregnancy, with an important psychological impact on mothers, necessitating precise identification and careful postpartum follow-up (1–3). In high-income countries, advanced maternal age, maternal smoking, obesity and primiparity are well-known risk factors of fetal demise (4). Etiology of stillbirth include placental anomalies and/or associated lesions, chromosomic, genetic, infectious, and inflammatory causes (2, 5–7). The wide spectrum of etiologies accounting for fetal demise, requires accurate clinical history taking, laboratory tests, ultrasound assessment, and most importantly pathological evaluation of the fetus and placenta (2, 5, 6). Among placental causes, vascular insufficiency inducing maternal vascular malperfusion (MVM), fetal vascular malperfusion (FVM) and increased peri-villous fibrin (IPF) remain an important cause of fetal anoxia and death (8, 9). In some cases, vascular insufficiency is associated with maternal thrombophilia, such as factor V Leiden mutation, especially in the context of recurrent pregnancy loss (10, 11). In some cases, umbilical cord anomalies can be the single explanation accounting for fetal IUGR, acute fetal asphyxia and stillbirth (8, 12, 13). Among umbilical cord abnormalities, the presence of a single umbilical artery (SUA), umbilical knots (UK), hypercoiled umbilical cord (HUC), umbilical cord thrombosis (UCT) or umbilical vessel aneurysm (UVA) account for most of the etiologies of IUGR and stillbirth in developing fetuses (12–15). Umbilical vessel aneurysms remain very rare yet potentially lethal abnormalities of the umbilical cord, especially in association with disturbed blood flow, aneurysm rupture, or intra-vascular thrombosis (15–17). In this article, we report a unique case of umbilical artery aneurysm thrombosis in a mother suffering from thrombophilia (factor V Leiden mutation) leading to stillbirth at 38 weeks of amenorrhea (WA) in an otherwise healthy woman.

## 2. Case description

**Figure 1:**
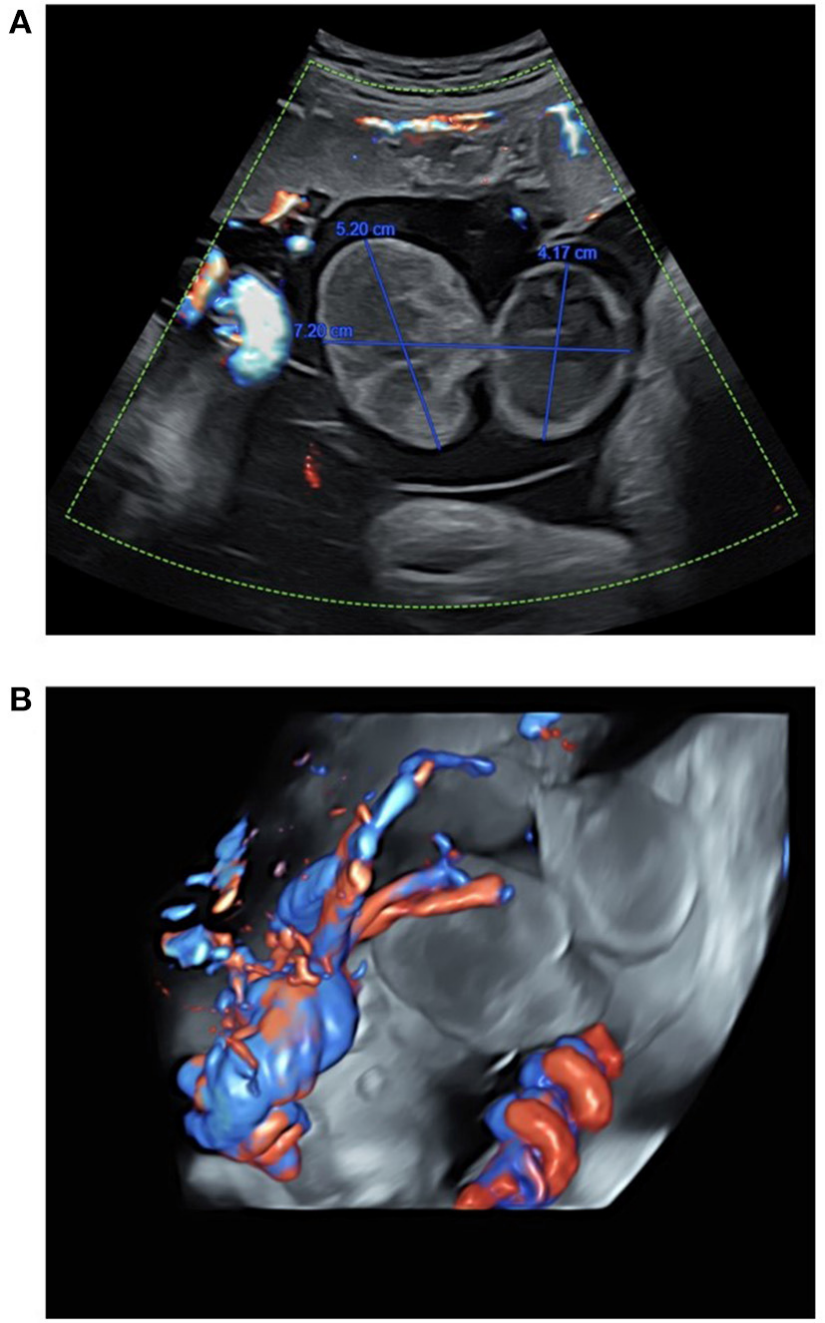
Ultrasound assessment of the umbilical cord in a 29-year-old mother at 37 WA, before stillbirth. **(A)** Ultrasound showed a bilocular cyst measuring 72 mm of major axis, and 31–52 mm of minor axis. **(B)** Umbilical vessel assessment of blood flow using tridimensional high definition Doppler ultrasound showing blood flow around the umbilical cord cyst.

A 29-year-old Caucasian Gravida 2 Para 0 mother admitted to the Department of Obstetrics and Gynecology (University Hospital of Dijon—France) for the absence of fetal movement at 38 WA. Past medical history includes previous early miscarriage associated with previous maternal smoking. The mother was not under medication during pregnancy. Maternal body mass index was otherwise normal (23.8 kg/m^2^). Concerning family history, the patient's mother and grandmother suffered from recurrent thrombophlebitis. Maternal serologies remained negative (mother naive for toxoplasmosis and viral infections) except for elevated IgG against rubella virus. First-trimester maternal serum screening was otherwise normal, with free β-human chorionic gonadotrophin (free β-hCG) at 39.200 IU/L −1,2 Multiple of the Median (MoM), pregnancy associated plasma protein A (PAPP-A) at 3.77 IU/L −1.07 MoM, and nuchal translucency at 1.3 mm −0.83 MoM. Combined first-trimester screening for trisomy 21 remained beyond 1/10.000, which indicated the absence of fetal aneuploidy. During pregnancy, first-trimester (12 WA) and second-trimester routine ultrasounds (22 WA) were otherwise normal. Third-trimester ultrasound performed at 37 WA revealed a 73 mm (major axis) cystic heterogenous mass at the umbilical cord insertion (Figure 1). The patient was referred to the Prenatal Diagnostic Center of the University Hospital of Dijon for further investigation. Ultrasonography performed at the Prenatal Diagnostic Center at 37 WA confirmed the presence of the cystic mass at the umbilical cord insertion, which revealed normal blood flow. The mother was discharged from the hospital, with appropriate instructions in case of abnormal fetal movements and/or signs of labor. The mother was later admitted to the Gynecology Emergency Services (University Hospital of Dijon) at 38 WA for abdominal pain and absence of fetal movements. Ultrasonography confirmed the absence of fetal movements and fetal cardiac activity. At time of fetal death, maternal blood testing was performed to rule out coagulation disorders or associated infection. The Kleihauer and antiglobin test were negative, indicating the absence of fetal-maternal hemorrhage or fetal hemolytic anemia. Elevated C-Reactive protein (20.3 mg/L) was associated with hyperleukocytosis (18.5 G/L), thus raising suspicion for chorioamnionitis. Immune assays revealed positive anti-nuclear antibodies (titers 1/160). Fibrinogen (3.3 g/L) and prothrombin factors (factor II: 112%, factor V: 90%, factor VII: 90%) were within normal ranges. Testing for SARS-CoV-2, CMV and HSV infections were negative at time of fetal death.

**Figure 2:**
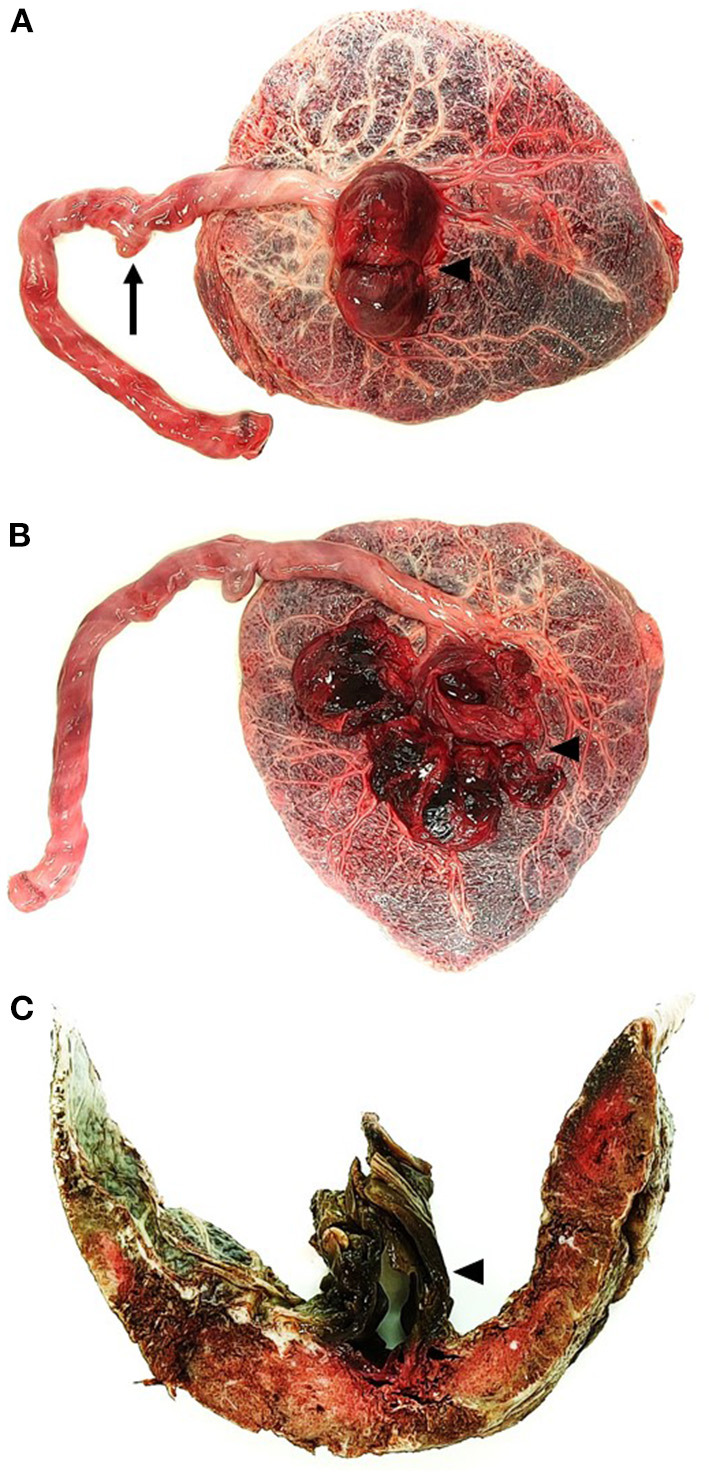
Gross examination of the placenta of a 29-year-old with stillbirth at 38 WA. **(A)** Placental examination of the fetal plate revealed a false knot on the umbilical cord (arrow) associated with a large aneurysmal cyst at the insertion of the umbilical cord (arrowhead). **(B)** At dissection, the aneurysmal cyst was filled with large blood clots (arrowhead). **(C)** At cut-section, the placental parenchyma showed subchorionic whitish nodules and confirmed the presence of the aneurysm, in relation to the umbilical arteries (arrowhead).

**Figure 3:**
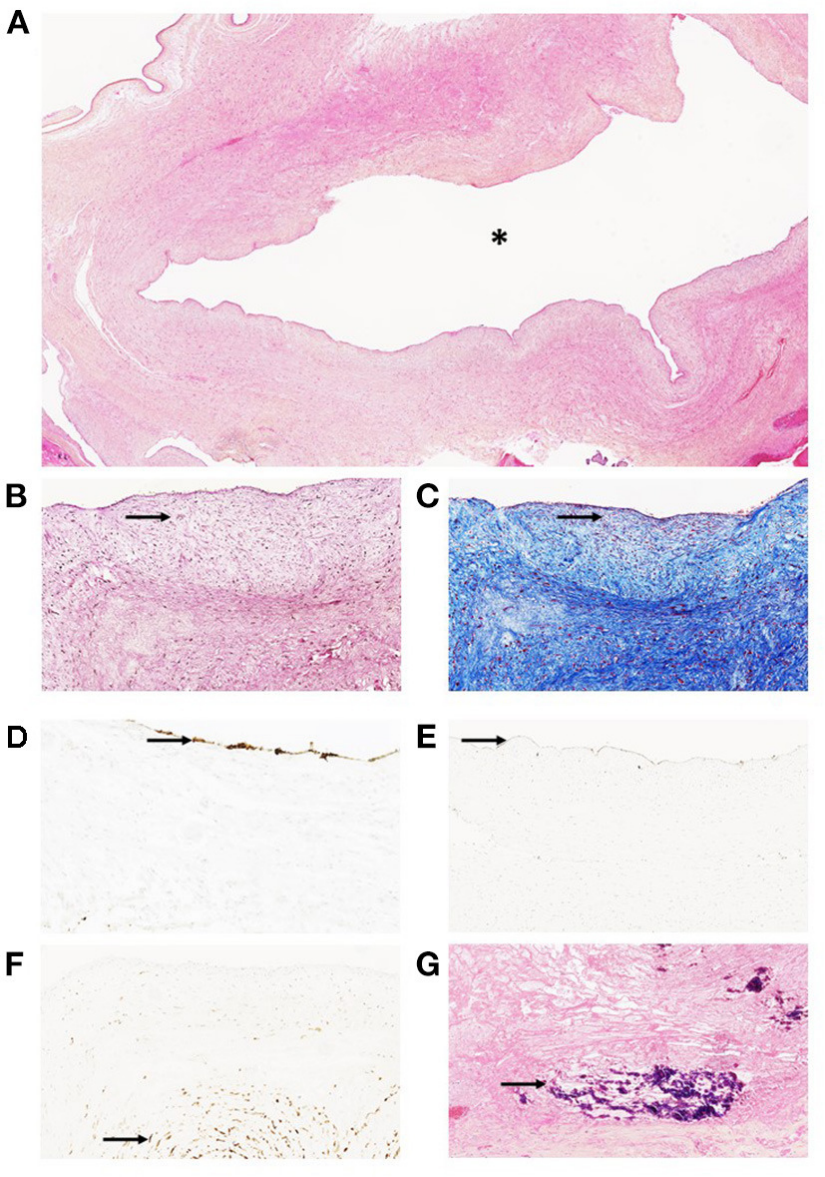
Histopathological analysis of the aneurysmal cyst of the umbilical cord in a 29-year-old mother with stillbirth at 38 WA. **(A)** (Hematoxylin Eosin Saffron—HES, × 100): histopathology showed a thick-walled large cyst (asterisk). **(B)** (HES, × 200)–**(C)** (Trichrome Blue, × 200): The wall of the cyst is mainly composed of mesenchymal cells and few scattered smooth muscle cells (arrows). **(D)** (CD34, × 200): Immunodetection of CD34 revealed few endothelial cells at the surface of the cyst (arrow). **(E)** (D2-40, × 200): Few mesenchymal cells exhibited cytoplasmic positivity after immunodetection (arrow). **(F)** (CD31, × 200): immunodetection of CD31 revealed few endothelial cells at the surface of the cyst (arrow). **(G)** (HES, × 200): calcified hemorrhagic debris (arrow) were seen after dissection and sampling of the aneurysmal cyst.

**Figure 4:**
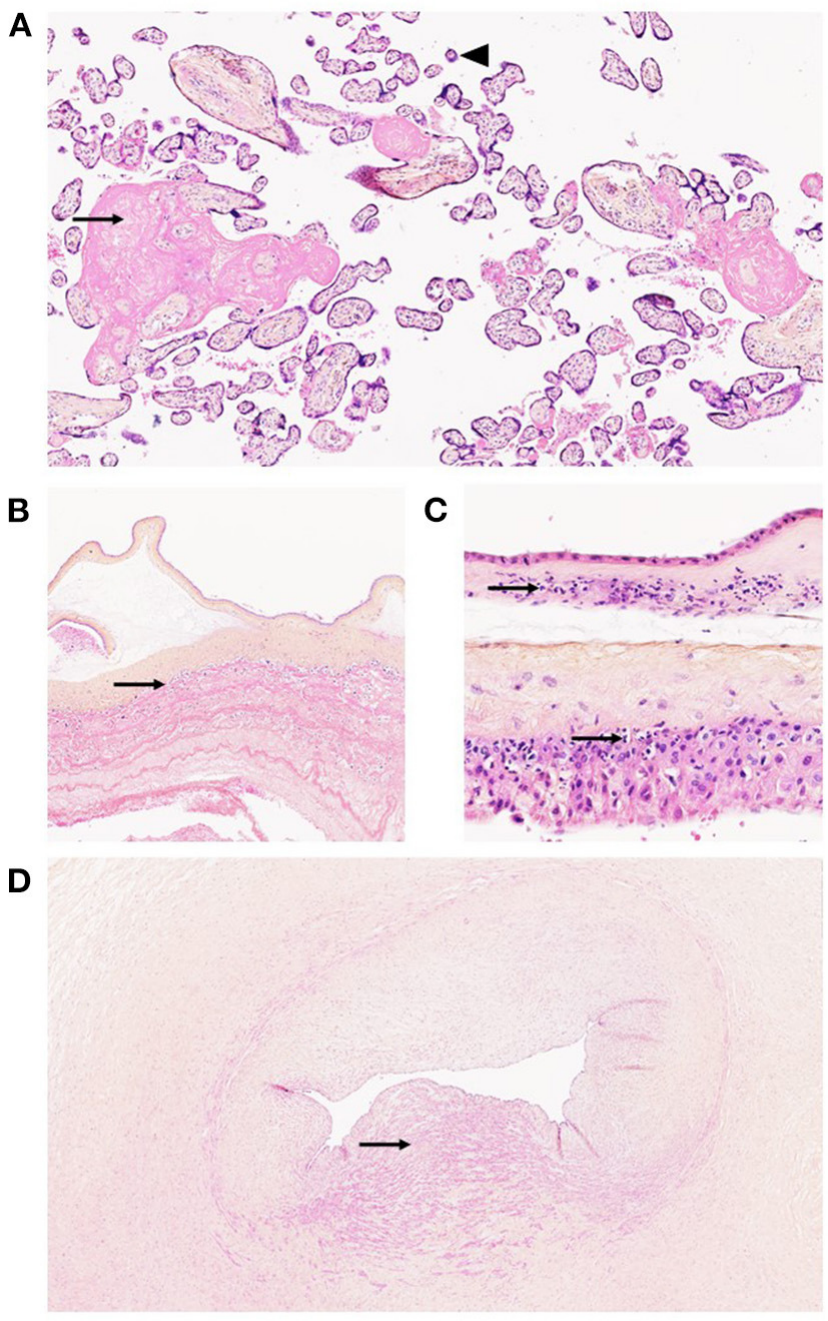
Histopathological analysis of the placenta in a 29-year-old mother with stillbirth at 38 WA. **(A)** (HES, × 200): Increased perivillous fibrin (arrow) and distal villous hypoplasia (arrowhead) were seen, indicating maternal vascular malperfusion. **(B)** (HES, × 200): Subchorionic thrombosis was observed (arrow), characterized by linear deposition of fibrin and red blood cells in the intervillous space adjacent to the fetal plate. **(C)** (HES, × 400): Histopathology showed evidence of stage 1 grade 2 chorioamnionitis, characterized by neutrophilic infiltration of the decidua parietalis of the membranes (arrows), without amniotic necrosis. **(D)** (HES, × 200): Histopathology showed rarefaction of smooth muscle cells (arrow) at the level of umbilical vessels adjacent to the aneurysmal cyst, inserted at the fetal plate.

Vaginal delivery and fetal expulsion were then performed. After delivery, the placenta and the fetus were referred to the Department of Pathology (University Hospital of Dijon) for further analysis. Management of stillbirth was performed according to the 2016 French guidelines (18). Placental analysis was performed in accordance with the 2016 Amsterdam consensus (9). Fetal autopsy was performed according to the current French guidelines (19). At autopsy, fetal pathology revealed a non-macerated, female eutrophic fetus (weight: 2,952 g −40th percentile), showing cyanosis of the lips and fingers, devoid of dysmorphic traits (including facial dysmorphism or limb anomalies) (20). Fetal measurements were within normal ranges, including crown-heel length (49 cm −40th percentile), crown-rump length (33 cm −30th percentile) and head circumference (33 cm −50th percentile) (21). At dissection, formalin-fixed organ weights remained within normal ranges, without evidence of malformation (22). Histopathological analysis revealed, apart from diffuse visceral congestion, meconial and keratin pigments in lung alveoli, thus suggesting previous meconium aspiration syndrome in the context of acute fetal distress. Fetal karyotype performed using thymic tissue was normal (negative for aneuploidy or chromosomal anomalies), with a 46 XX formula. Gross examination of the placenta revealed a eutrophic placenta (496 g −50th percentile), of normal configuration (oval shape), measuring 24 cm of length, 18 cm of width and 2 cm of thickness, with normal membrane insertion (23, 24). The umbilical cord measured 36 cm of length and 2 cm of diameter. At the fetal plate, a large bilocular cystic lesion was observed at the insertion of the umbilical cord (Figure 2) measuring ~8 cm of diameter. At dissection, the cystic lesion was filled up with hemorrhagic debris and large blood clots (Figure 2). Placental cut section revealed whitish subchorionic nodules compatible with subchorionic thromboses (SCT; Figure 2). Histopathological analysis of the cystic lesion revealed a large cavity filled with hemorrhagic debris at the base of the umbilical cord (Figure 3). The wall of the cyst was mainly composed of elastic fibers intermingled with scattered smooth muscle cells (Figure 3). Few scattered CD34^+^ CD31^+^ D2-40^+^ endothelial cells were observed close to the lumen of the cyst (Figure 3). Histopathological analysis of the subchorionic nodules confirmed SCT (Figure 4). Concerning the placental villi, MVM and focal IPF were also observed (Figure 4). Histopathological analysis of the membranes revealed stage 1 grade 2 chorioamnionitis (Figure 4). Furthermore, histopathological analysis showed rarefaction of smooth muscle cells at the level of umbilical vessels adjacent to the aneurysmal cyst (Figure 4).

Considering placental pathological examination (MVM, SCT, IPF, aneurysmal thrombosis), previous stillbirth, and previous family history of thromboembolism, the mother was referred to the Department of Hemostasis (University Hospital of Dijon—France) for thrombophilia testing. Blood analysis indicated an antithrombin activity within normal ranges (122%). The protein C resistance test revealed an increased coagulation time of maternal blood with adjunction of activated C protein (35 s before adjunction, vs. 62.9 s after adjunction of activated C protein). Protein S activity was measured at 70% of normal activity. Lupus anticoagulant testing was negative. Further genetic analysis of the maternal blood revealed the presence of heterozygous factor V Leiden mutation (c.1691G>A; p.Arg506Gln), which confirmed thrombophilia (LightCycler^®^ 480 System, Roche—Switzerland). After the episode, the presence of maternal thrombophilia would indicate the necessity for preventive anticoagulant therapy (100 mg of aspirin per day) during pregnancy, associated with low-molecular weight heparin (LMWH) for 6 weeks postpartum.

## 3. Discussion

Umbilical artery aneurysms remain a very rare yet lethal finding in the placenta, with only six live births (16, 25, 26). To date, including our case, only 18 cases were reported in the literature (16). Two thirds (12/18) of the published cases were associated with a single umbilical artery, and one quarter (4/18) with placental trisomy 18 mosaicism (17, 26–32). The pathophysiology of UAA might be explained by the increased weakness of umbilical arteries at their insertion on the fetal plate, where Wharton's jelly is relatively less abundant, thus favoring the appearance of aneurysms (16, 25, 28). The presence of an increased fetal cardiac output during development might explain the increase in umbilical artery intravascular pressure and the genesis of an aneurysm, in areas of greater elasticity where Wharton's jelly is absent (25, 26, 28). Including our case, 12 out of 18 UAA were located at the insertion of the umbilical cord (16, 25, 28). However, in our case, no evidence of trisomy mosaicism or single umbilical artery was noted.

In all cases of UAA, the cystic appearance of UAA during ultrasonography routine checkups can potentially lead to a misdiagnosis of a non-lethal umbilical cord pseudocyst, patent urachus or omphalocele (29, 33–35). Current guidelines for management of umbilical cord cysts in the second and third trimester imply to perform fetal karyotype testing in order to rule out aneuploidy, due to the frequent association of umbilical cord cysts and chromosomal anomalies such as trisomy 13 or 18 (29, 33). Nevertheless, in the absence of chromosomal anomalies, the presence of a potentially lethal vascular malformation of the umbilical cord should be considered. The potential lethality of such rare lesions of the umbilical cord should raise awareness for the discussion of new up-to-date guidelines on the management of umbilical cord cystic lesions in otherwise healthy fetuses. Scheduled induction of labor and preventive anticoagulant therapy should therefore be considered in large umbilical cord cysts, regardless of the presence of reassuring signs at ultrasonography.

In our case, history of previous stillbirth motivated thrombophilia testing in this patient. Previous studies showed that mothers carrying Factor V Leiden mutation had higher rates of early and late fetal loss during pregnancy (36–38). Histopathological findings in placentas of mothers with thrombophilia, including placental infarcts, MVM, IPF, and avascular villi, provide a partial explanation for chronic placental malperfusion, IUGR, fetal hypoxia and fetal demise (39, 40). In our case, the presence of MVM and IPF could be attributed to maternal Factor V Leiden mutation, without IUGR (eutrophic fetus-−40th percentile). The presence of UAA alone provides an explanation for fetal demise, as very high rates of stillbirth in mothers carrying UAA were observed in the literature. Of note, the occurrence of intra-aneurysmal thrombosis remain poorly explained in the literature. Data concerning UAA showed that compression of surrounding umbilical vessels following the formation of a large-sized aneurysm could lead to vascular thrombosis following altered blood flow (27, 41). In our case, we can hypothesize that the presence of maternal thrombophilia might have facilitated aneurysm thrombosis and acute fetal asphyxia. Few studies have focused on the possible outcomes of umbilical artery thrombosis, which remain a very rare event during pregnancy (42, 43). The association of umbilical artery thrombosis with Factor V Leiden mutation remain controversial in the literature and poorly described (42, 43). Without enough clear evidence of the association between maternal Factor V Leiden mutation and umbilical artery thrombosis, further studies will be required in order to explore putative links between UAA and thrombophilia. As an example, polymorphisms of the angiotensin-converting enzyme gene, involved in preeclampsia, have been demonstrated as risk factors of aneurysm formation and potentially identified as a cause of thrombophilia (44–46).

## Data availability statement

The original contributions presented in the study are included in the article/supplementary material, further inquiries can be directed to the corresponding authors.

## Ethics statement

Ethical approval was not provided for this study on human participants because patient consent was obtained for the case report. The patients/participants provided their written informed consent to participate in this study.

## Author contributions

Writing and editing: GT, CO, and LM. Resources: ED, FH, OM, NI, LR, and LM. Investigation: GT, CO, ED, OM, NI, and LR. All authors contributed to the article and approved the submitted version.
